# Transcriptomic reveals key genes and regulatory pathways in galactomannan biosynthesis in *Gleditsia sinensis* polysaccharide gum

**DOI:** 10.3389/fpls.2025.1555374

**Published:** 2025-07-23

**Authors:** Yanyan Zhang, Tingting Yang, Xiangpei Wang, Feng Xu, Linlin Du, Hongmei Wu

**Affiliations:** ^1^ College of Pharmacy, Guizhou University of Traditional Chinese Medicine, Guiyang, China; ^2^ School of Ethnic Medicine, Guizhou Minzu University, Guiyang, China

**Keywords:** *Gleditsia sinensis* Lam., galactomannan, transcriptome, biosynthesis, unigenes enrichment analysis

## Abstract

**Introduction:**

The *Gleditsia sinensis* Lam. polysaccharide gum contained in *G. sinensis* seeds is not only an additive specified in national food standards, but also an important strategic resource for industrial raw materials such as oil and natural gas in China.The main component of *G. sinensis* polysaccharide gum is galactomannan (GM). To date, most studies have focused on the structural modification and component separation of the polysaccharide gum of *G. sinensis* seeds, with only a few reports on the regulatory genes involved in its formation.

**Methods:**

Transcriptome analysis was performed to assess the gene networks associated with GM synthesis in *G. sinensis* seeds at four stages: 6, 9, 12, and 16 weeks after flowering.

**Results:**

The result indicated that differential expression analysis identified 20 unigenes linked to five critical enzymes in the GM biosynthesis pathway. Further pathway enrichment analysis demonstrated that fructokinase, galactose gyrase, inositol galactoside synthase, phosphogalactosyltransferase, and raffinose synthase play pivotal roles in GM biosynthesis, positively regulating its production.

**Discussion:**

The results of this study provide new ideas for the research of GM biosynthesis related genes in *G. sinensis* and enhance the potential application prospects in genetic engineering.

## 1 Introduction

Dried and mature seeds of leguminous plant *Gleditsia sinensis* Lam., also known as Zaojiazi (Liu and Zhao, 2009), are warm and exhibit a pungent taste, aligning with the lung and large intestine meridians in traditional Chinese medicine. These seeds exhibit various pharmacological benefits, including proper moisturization, promotion of bowel movements, dispelling of wind, nodule dispersion, and swelling reduction. Consequently, they are used to treat various symptoms and conditions, such as constipation, urgent diarrhea with tenesmus, hernia pain, scrofula, swelling, toxicity, and skin diseases. They are listed in the local standards of traditional Chinese medicine in the Shandong and Henan provinces (Hao et al., 2012; Li and Zhang, 2018). Polysaccharide gum derived from *G. sinensis* seeds is used as an additive according to the national food standards and an industrial raw material for oil and natural gas, serving as an important resource in China (Li et al., 1980; Zhang and Xiao, 1990; Shao and Yuan, 2005; Jiang et al., 2009; Liu, 2022). The main component of *G. sinensis* polysaccharide gum is galactomannan (GM), which is composed of (1-4) β-D-pyranose mannose groups as the backbone and (1-6)-linked α-D-pyranose galactose residues as the side chains. *G. sinensis* polysaccharides are composed of mannose, glucose, and galactose (Wu et al., 2025). To date, most studies have focused on the structural modification and component separation of the polysaccharide gum of *G. sinensis* seeds, with only a few reports on the regulatory genes involved in its formation (Lei et al., 2018; Guo, 2020). Identification of the key regulatory genes in the GM synthesis pathway of *G. sinensis* seeds will facilitate the enhancement of GM content via genetic engineering, thereby enhancing the quality and yield of *G. sinensis* polysaccharide gum. This study aimed to establish a solid foundation for *G. sinensis* molecular breeding. In this study, we conducted transcriptome analysis of *G. sinensis* seeds at different growth stages to examine the relationships between their growth and developmental stage and GM biosynthesis-related functional gene levels and explore the underlying regulatory mechanisms. This study provides basic information for future research on GM biosynthesis-related gene functions during *G. sinensis* seed development.

## 2 Materials and methods

### 2.1 Plant materials and sampling

Zaojiazi is the dried and mature seed of leguminous plant *Gleditsia sinensis* Lam., *G. sinensis* Lam. Seeds were collected from various locations in Guizhou Province, China. Representative distribution areas of *G. sinensis* in Guizhou: covering karst mountainous areas (Guanling Town), hilly basins (Dangwu Town), plateau plateaus (Jichangba) and other habitats. In southern Guizhou, Lushan Town receives annual precipitation up to 1,300 mm, while in the northwestern Bijie City, rainfall is only 900–1,100 mm. Such humidity variations may significantly affect seed germination rates. Yellow earth (around Guiyang, acidic, low phosphorus), Limestone soil (Guanling Town, Anshun, high calcium content, karst region), Purple soil (Jinbi Town, Qianxi City, relatively fertile). Despite the small geographic scale, Guizhou’s complex topography and microclimates create distinct environmental gradients, affecting the accumulation of *G. sinensis* seeds GM. Based on the post-flowering period, their growth and developmental stages were categorized into four types: 6, 9, 12, and 16 weeks post-flowering stages. The 6, 9, 12, and 16 weeks post-flowering stages were selected based on the biological characteristics and physiological and biochemical changes during *G. sinensis* seed development, according to our research objectives. These time points provide systematic and in-depth developmental dynamic information, facilitating the evaluation of the regulatory patterns underlying *G. sinensis* seed development (McCleary et al., 1987; Li et al., 2018). Ten samples (**Table 1**) from Guizhou Province were tracked and sampled across the four growth stages. The seeds were confirmed as *G. sinensis*. seeds by Professor Wang Xiangyu at Guizhou Minzu University. To obtain *Gleditsia* polysaccharide gum (GPG) for GM content determination, endosperms of seeds at different growth stages were extracted, dried, and pulverized. Fresh seeds were wrapped in aluminum foil, appropriately labeled, stored in liquid nitrogen, and transferred to a –80 °C freezer for long-term preservation. These frozen seeds were used for subsequent RNA extraction and transcriptome sequencing.

**Table 1:** Sample sources.

Sample	Source	Latitude and longitude	Sample	Source	Latitude and longitude
QG	Huaxi District, Guiyang City	26°22′59″N 106°37′18″E	DJ	Zhijin County, Bijie City	26°31′53″N 106°01′12″E
QY	Qingyan Town, Guiyang City	26°19′53″N 106°41′23″E	YD	Yuduo Town, Qianxi City	26°53′39″N 106°01′20″E
GP	Gaopo Township, Guiyang City	26°16′50″N 106°51′25″E	JB	Jinbi Town, Qianxi City	26°56′44″N 105°58′19″E
MC	Machang Town, Bijie City	26°38′54″N 106°5′50″E	GL	Guanling Town, Anshun City	25°57′12″N 105°37′18″E
MJ	Chicken Farm Dam in Bijie City	26°30′59″N 106°01′39″E	LS	Lushan Town, Qiannan Prefecture	25°56′34″N 106°30′03″E

### 2.2 Experimental instruments and reagents

The following instruments and reagents were used in this study: KZ-III-F High-speed Low-Temperature Tissue Grinder (Wuhan Sevier Biotechnology Co., Ltd.), SYQ-DSX-280B Portable Stainless Steel Pressure Steam Sterilization Pot (Shanghai Shen’an Medical Equipment Factory), Bio Rad Electrophoresis Tank and DYY-6C Electrophoresis Instrument (Beijing Liuyi Instrument Factory), Bio Rad Gel Imaging System XRS^+^ and MK3 Full Function ELISA Reader (Thermo Fisher Instrument Co., Ltd.), Waters-e2695 HPLC Chromatograph (Waters Corporation, USA), DW-86L386 Ultra-Low Temperature Refrigerator (–80 °C; Haier Group), N60 Ultra Micro Spectrophotometer (IMPLEN GmbH, Germany), Plant RNA Extraction and PCR kits (Tiangen Biochemical Technology Co., Ltd.), and BCD-248-20 °C/4 °C Refrigerator (Electrolux Corporation).

### 2.3 Determination of GM levels

#### 2.3.1 Sample pretreatment

Endosperms were stripped from the *G. sinensis* seed samples, dried, powdered, and sieved through a No. 5 sieve to obtain the *G. sinensis* polysaccharide gum. According to the current standard GB/T 31742-2015 of the People’s Republic of China, 300 mg ± 10 mg of GPG was accurately weighed and placed in a pressure-resistant bottle. Then, 3 mL of 72% sulfuric acid was added, and the mixture was stirred with a glass rod for 1 min. After uniform dispersion, the mixture was placed in a constant temperature water bath at 30 °C for 60 min. Subsequently, 85 mL of deionized water was added to dilute the sulfuric acid to 4%. The pressure-resistant bottle was tightly sealed and placed in an autoclave at 121 °C for 60 min. After removal and cooling, calcium carbonate was added to neutralize the solution and achieve a pH of 5–6. The mixture was centrifuged at 4000 rpm to obtain the supernatant, which was the hydrolyzed sample of GPG.

#### 2.3.2 Preparation of the reference solution

Appropriate amounts of the four monosaccharide references were accurately weighed and dissolved in distilled water to prepare the mixed reference solution with glucose, galactose, *D*-mannose, and *D*-xylose concentrations of 2.175, 11.900, 21.780, and 2.418 mg/mL, respectively. This solution was stored at 4 °C for future use.

#### 2.3.3 Preparation of the test solution

Pretreated hydrolyzed sample of GPG or reference solution (1 mL) was placed in a test tube, and 1 mL of 0.3 mol/L NaOH solution was added. After mixing, 1 mL of 0.5 mol/L 1-phenyl-3-methyl-5-pyrazolone-methanol solution was added. The mixture was thoroughly mixed and placed in a water bath at 70 °C for 1 h. After cooling, 1 mL of 0.3 mol/L HCl solution was added to neutralize NaOH. After adding 1 mL of chloroform, the solution was vortexed for 30 s and centrifuged. The chloroform layer was discarded. This extraction process was repeated twice to ensure the complete removal of excess 1-phenyl-3-methyl-5-pyrazolone. Finally, the solution was filtered through a 0.22-µm microporous membrane to obtain the test solution.

#### 2.3.4 Measurement of GM levels

Diamononsil C18 chromatographic column (250 mm × 4.6 mm; 5 μm) was used for chromatography. The mobile phase consisted of acetonitrile (A) and a 0.05 M ammonium acetate solution (B) at a flow rate of 1 mL/min. Injection volume was 10 μL, detection wavelength was 254 nm, and column temperature was 40 °C. Chromatographic separation was performed according to the mobile phase gradient shown in **Table 2**, with an analysis time of 42 min.

**Table 2:** Mobile phase gradient elution table.

Time (min)	Mobile phase A	Mobile phase B
0–2	18%	82%
2–12	18–20%	82–80%
12–32	20–22%	80–78%
32–35	22%	78%
35–42	22–40%	78–60%

### 2.4 Transcriptome sequencing and data analysis

#### 2.4.1 RNA extraction and quality assessment

In total, 12 samples were collected from three biological replicates at each QY1–4 stage. Total RNA was extracted from *G. sinensis* seeds, following the procedures outlined in the Plant RNA Extraction Kit. Then, OD260/OD280 ratio of RNA was measured using the multifunctional ELISA Reader. Here, 2 µL of RNA solution was used to assess its purity. Subsequently, RNA concentration and quality were assessed using the Agilent 5300 Capillary Electrophoresis system.

#### 2.4.2 Quality control of the sequencing data

Qualified RNA samples were subjected to transcriptome sequencing using the Illumina HiSeq 4000 platform. The sequencing image signals were converted into text signals using CASAVA base calling on the Illumina HiSeq 4000 platform and stored in fastq format as raw data. Raw reads were subjected to quality control using the SeqPrep and Sickle tools to remove the adapter sequences, trim the low-quality bases, discard the reads containing N bases, and eliminate the sequences shorter than 30 bp after trimming. High-quality clean reads were obtained after quality control to ensure the accuracy of subsequent analyses. Trinity software (Fang et al., 2022) was used to cluster the clean reads into unigenes.

#### 2.4.3 Unigene annotation and analysis

TopHat software (Trapnell et al., 2009) was used to align the quality-controlled read data to obtain the mapped reads. The mapped reads were further assembled into transcripts. Basic Local Alignment Search Tool (Ashburner et al., 2000), with *E*-value ≤ 10-5, was used to annotate the transcripts against six databases: Non-Redundant (NR), Gene Ontology (GO), Evolutionary Genealogy of Genes: Non-Supervised Orthologous Groups (eggNOG), Kyoto Encyclopedia of Genes and Genomes (KEGG), Protein Family, and Swiss-Prot databases (Tatusov et al., 2000; Koonin et al., 2004; Kanehisa et al., 2004; Trapnell et al., 2010; Xie et al., 2011; Huerta-Cepas et al., 2016). Comprehensive annotation information of the transcripts was obtained, and annotation status in each database was statistically summarized.

#### 2.4.4 Differential gene expression analysis

Bowtie (McKenna et al., 2010) was used to align the sequencing reads and unigene data. The alignment results were further analyzed using the RSEM software (Li and Dewey, 2011) to estimate the expression levels, which were represented as the transcripts per million (TPM) values of the corresponding unigenes. Differential gene expression analysis was conducted using the DESeq2 software (Anders and Huber, 2010). Number of differentially expressed genes (DEGs) was counted using false discovery rate < 0.01 and | log2 (FC) | ≥ 2 as the screening criteria. Moreover, Benjamini–Hochberg correction was applied for multiple testing adjustments.

### 2.5 Statistical analyses

GM content data of various samples were imported into SPSS 26.0 and SIMCA-P 14.0 for comprehensive statistical analyses.

## 3 Results

### 3.1 Determination of GM levels

Samples were prepared by processing the polysaccharide gum of *G. sinensis* collected at different seed growth stages. Each sample was injected three times consecutively, and the peak areas of *D*-mannose and galactose were recorded. These peak areas were substituted into the corresponding linear regression equations to calculate the proportion of GM in the *G. sinensis* polysaccharide gum (**Table 3**). *D*- mannose: Y=1963.2x+269193, R^2^ = 0.9994, 2.178~0.218mg/mL; galactose: Y=19896.3x-47828, R^2^ = 0.9997, 1.19~0.119mg/mL.

**Table 3:** GM levels in *Gleditsia sinensis* seeds (percentage of dry weight) (n = 3).

Sample	6 weeks (%)	9 weeks (%)		12 weeks (%)	16 weeks (%)
MC	43.78 ± 0.19	46.33 ± 0.52		50.22 ± 1.26	52.25 ± 0.23
QG	43.32 ± 0.46	46.87 ± 0.154		50.78 ± 2.48	52.12 ± 0.15
GP	46.24 ± 0.147	47.24 ± 0.12		51.45 ± 0.47	53.32 ± 0.23
MJ	46.96 ± 0.64	48.31 ± 1.46	51.63 ± 1.11		55.99 ± 2.48
DJ	47.52 ± 0.73	49.63 ± 0.23	50.74 ± 0.68		51.93 ± 0.76
QY	46.31 ± 0.37	48.24 ± 0.56	52.52 ± 0.39		54.11 ± 1.12
YD	45.24 ± 0.31	48.43 ± 0.45	49.96 ± 0.44		52.01 ± 2.31
JB	41.21 ± 1.31	49.31 ± 0.78	51.63 ± 0.12		55.23 ± 1.13
GL	44.33 ± 0.18	48.54 ± 0.79	51.32 ± 0.22		54. 12 ± 0.54
LS	46.24 ± 0.12	47.37 ± 2.18	50.55 ± 0.18		53.84 ± 0.22

### 3.2 Orthogonal partial least-squares discriminant analysis

**Figure 1:**
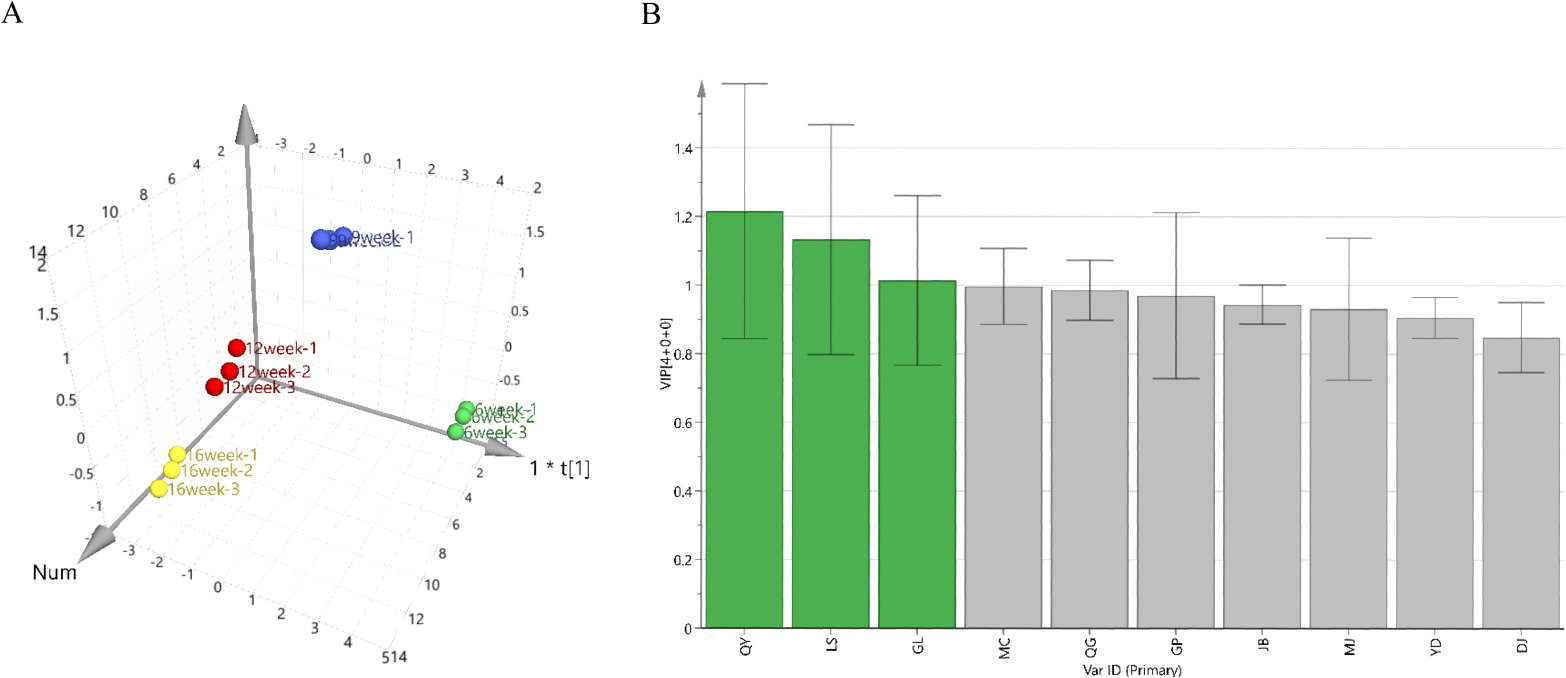
Orthogonal partial least-squares discriminant analysis (OPLS-DA). **(A)** OPLS-DA model diagram. **(B)** Differential variable importance in projection (VIP) value graph.

GM levels in *G. sinensis* seeds at four different growth stages were used as variables and imported into the SIMCA-P 14.0 software for orthogonal partial least-squares discriminant analysis. In this model, R^2^X and R^2^Y were 0.99 and 0.994, respectively, and Q^2^ was 0.943, all of which were greater than 0.5, confirming that the established model was stable and reliable. Differences in the GM levels of different growth stage samples are shown in **Figure 1A**. Samples with statistically significant differences were screened by ranking the variable importance in projection values in the model. As shown in **Figure 1B**, three samples (QY, LS, and GL) exhibited variable importance in projection values > 1. Based on these results, the four growth stages of *G. sinensis* seeds from the QY sample were selected for subsequent transcriptome analysis.

### 3.3 Sample RNA quality

RNA sample integrity was assessed via 1% agarose gel electrophoresis. RNA purity and concentration in each sample were examined using NanoDrop 2000. Furthermore, RNA quality number was determined using the Agilent 5300 system (**Table 4**). Notably, RNA quality and integrity of all *G. sinensis* seed samples were satisfactory, making them suitable for subsequent analysis.

**Table 4:** RNA quality of each sample.

Growth stage	Sample	Concentration (ng/µL)	Total (µg)	OD260/280	OD260/230	RQN
6 weeks after flowering	QY1_1 QY1_2 QY1_3	43.00 132.80 43.90	1.51 4.65 1.54	2.02 2.18 2.02	0.52 1.61 0.27	9.50 9.50 9.40
9 weeks after flowering	QY2_1 QY2_2 QY2_3	863.30 409.80 318.20	30.22 14.31 11.14	2.22 1.92 1.98	1.91 1.17 0.66	10.00 9.90 9.00
12 weeks after flowering	QY3_1 QY3_2 QY3_3	275.40 332.50 400.20	9.64 11.64 14.01	2.15 2.11 2.12	1.96 2.16 1.86	9.60 9.90 9.00
16 weeks after flowering	QY4_1 QY4_2 QY4_3	391.50 146.70 453.20	13.70 5.13 15.86	2.10 2.12 2.09	1.78 1.60 1.26	9.60 9.70 9.70

### 3.4 Overall transcriptome sequencing analysis

Qualified RNA samples were subjected to high-throughput sequencing using the Illumina HiSeq 4000 platform to obtain the gene expression profiles of *G. sinensis* seeds at different growth stages. After a series of data filtering and redundancy removal steps, clean data were obtained for 12 samples, totaling 79.13 GB (**Table 5**). Each sample had clean data exceeding 6.23 GB, with a GC content of 44.45–46.38%. The GC content of Q20 was 97.32–97.84% and Q30 was 92.62–93.72%. These data suggest that the sequencing quality of *G. sinensis* seeds was high, facilitating further analysis.

**Table 5:** Transcriptome sequencing data statistics.

Growth stage	Sample	Raw reads	Clean bases	Q20 (%)	Q30 (%)	GC content (%)
6 weeks after flowering	QY1_1	43785090	6347349041	97.67	93.42	46.38
	QY1_2	45215238	6543668377	97.84	93.72	45.76
	QY1_3	44082364	6371722046	97.59	93.19	45.96
9 weeks after flowering	QY2_1	45327434	6559340510	97.43	92.89	45.94
	QY2_2	43698966	6439518723	97.38	92.70	45.97
	QY2_3	44721014	6408948639	97.6	93.38	46.32
12 weeks after flowering	QY3_1	52426812	7604364374	97.32	92.62	45.51
	QY3_2	46650124	6805143966	97.58	93.13	46.08
	QY3_3	46011062	6702895575	97.49	92.99	45.34
16 weeks after flowering	QY4_1	42777012	42499950	97.78	93.58	44.45
	QY4_2	45088626	44666760	97.56	93.12	44.90
	QY4_3	43706666	43398880	97.70	93.41	44.59

Using the Trinity software, we performed *de novo* assembly of the clean data of all samples and conducted an optimized evaluation of the assembly results. Number of assembled unigenes was 110,141, and the number of transcripts was 199,142. Average N50 length was 1,425 bp; **Table 6**). Proportions of transcripts and unigenes with lengths ≥ 1,000 bp were 58.295 and 77.957%, respectively. Collectively, these findings indicate the high sequencing and assembly completeness of the samples.

**Table 6:** Transcript data assembly optimization results.

Type	Unigene	Transcript
Total number	110141	199142
Total base	88541260	204239563
Longest length (bp)	17663	17663
Shortest length (bp)	201	201
Average length (bp)	803.89	1025.6
N50 length (bp)	1425	1767
E90N50 length (bp)	2922	2285
Fragment mapped percent (%)	58.295	77.957
GC percent (%)	38.48	39.42

### 3.5 Functional annotation of unigenes

Unigenes and transcripts obtained from the assembly of the four developmental stage samples of *G. sinensis* seeds were annotated and compared against databases, including the GO, KEGG, and Swiss-Prot databases. Annotation results for all databases are presented in **Table 7**. In total, 214,031 unigenes and 537,031 transcripts were annotated.

**Table 7:** Unigene functional annotations in various databases.

Database	Unigenes	Transcript
GO	44378	104669
KEGG	18662	52005
eggNOG	41792	105217
NR	53262	123333
Swiss-Prot	31740	83647
Pfam	24197	68160
Sum	214031	537031

**Figure 2:**
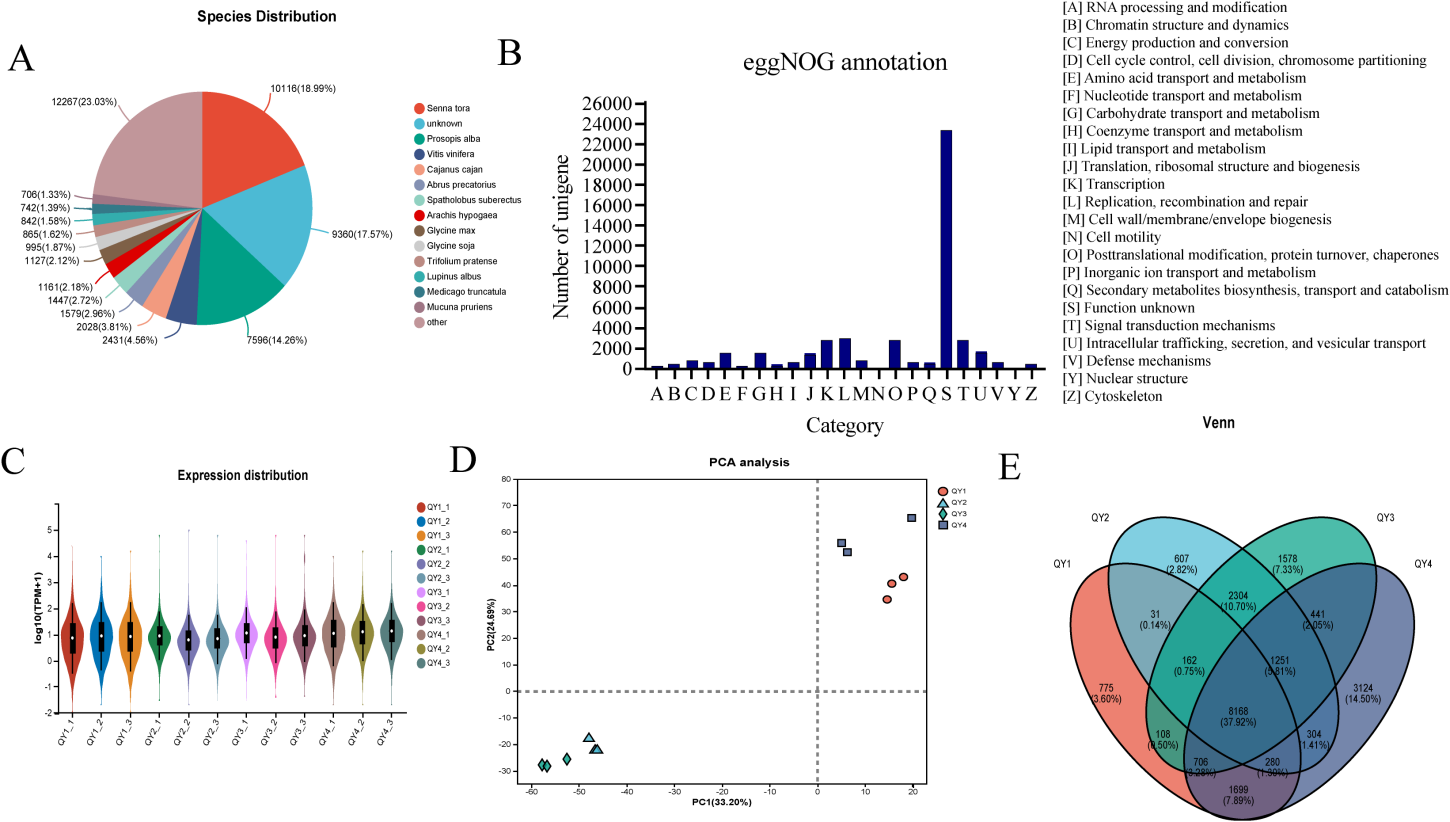
Functional annotation and expression profiling analysis of *G. sinensis* transcriptome data **(A)** Species distribution of NR-annotated genes; **(B)** Functional categorization by eggNOG annotation; **(C)** Expression level distribution across samples; **(D)** Principal Component Analysis (PCA) of sample relationships; **(E)** Comparative Venn diagram of expressed genes.

Specifically, 53,262 unigenes were annotated using the NR database. Among the species annotated in this database, *Senna tora*, *Prosopis alba*, *Vitis vinifera*, and *Cajanus cajan* ranked in the top four positions, accounting for 18.99, 14.26, 4.56, and 3.81% of all species, respectively (**Figure 2A**). GM accumulates extensively in the seed endosperm of many leguminous plants. Previous studies have also characterized the genes involved in GM biosynthesis in various leguminous plants, such as fenugreek (Wang et al., 2012) and guar (Naoumkina et al., 2007). This suggests that the GM synthesis pathway in *G. sinensis* is similar to that in other leguminous plants, providing a reference for subsequent transcriptome data analysis.

EggNOG database, which is rich in genomic information, was used to further investigate the functions of the identified genes via functional annotation, which resulted in 24 functional categories (**Figure 2B**). The category with the highest number of gene annotations was “[S] (function unknown),” encompassing 23,380 genes. This indicates the presence of a substantial number of genes with unknown functions in the *G. sinensis* seeds, warranting further exploration. The second largest category was “[L]: Replication, recombination and repair),” with 2,885 genes, followed by “[K]: Transcription” with 2,689 genes and “[T]: Signal transduction mechanisms” with 2,731 genes. Additionally, “[G]: Carbohydrate transport and metabolism” category included 1,453 genes. These results provide valuable insights into the GM biosynthesis pathway.

### 3.6 Gene expression analysis

Quantitative analysis of unigene expression levels was performed using the RSEM software. The number of transcripts was used as a unit, and TPM value served as a quantitative indicator to calculate the expression level of each gene. Logarithmic distribution of unigene expression levels across various samples is shown in **Figure 2C**. Data of different groups were relatively similar, suggesting that the gene expression patterns were similar among all groups.

Principal component analysis of 12 samples was conducted based on the gene expression levels. The first principal component accounted for 33.20%, whereas the second principal component accounted for 24.69% of the variance in the dataset (**Figure 2D**). This result suggests that different growth stage samples clustered together, indicating the reliability and effectiveness of biological replication in the selected samples.

Next, Venn diagram analysis of the four developmental stage samples was performed based on the TPM values (**Figure 2E**). Uniquely expressed genes were identified in each stage (QY1, QY2, QY3, and QY4), resulting in a total of 6,084 NR genes. Additionally, 8,168 common genes were expressed in all stages (QY1–4). These genes possibly play regulatory roles in the growth and development of *G. sinensis* seeds.

### 3.7 DEG screening

To investigate the dynamic changes in gene expression levels during *G. sinensis* seed development, we used the DESeq2 software based on the TPM values to conduct pairwise comparisons of the expressed genes in the four growth stage samples of *G. sinensis* seeds. The number of DEGs was counted using false discovery rate < 0.01 and | log2 (FC) | ≥ 2. Between QY1 and QY2, 31,655 DEGs, with more downregulated genes (22,036; 69.61%) than upregulated genes (9,619; 30.39%), were identified. Between QY1 and QY3, 33,548 DEGs, including 32.31% upregulated and 67.69% downregulated genes, were identified. Between QY1 and QY4, 8,252 DEGs, including 29.52% upregulated genes, were identified. Between QY2 and QY3, 5,342 DEGs, including 59.51% upregulated genes, were detected. Between QY2 and QY4, 22,016 DEGs, including 64.41% upregulated genes were identified, Between QY3 and QY4, 29,101 DEGs, including 60.18% upregulated genes, were noted (**Table 8**). In total, 57,767 upregulated and 72,147 downregulated genes were identified.

**Table 8:** List of differentially expressed genes at different growth stages *G. sinensis* seeds.

DEG Set	All DEG	Upregulated	Downregulated
QY1_vs_QY2	31655	9619	22036
QY1_vs_QY3	33548	10839	22709
QY1_vs_QY4	8252	2436	5816
QY2_vs_QY3	5342	3179	2163
QY3_vs_QY4	29101	17513	11588
QY2_vs_QY4	22016	14181	7835

**Figure 3:**
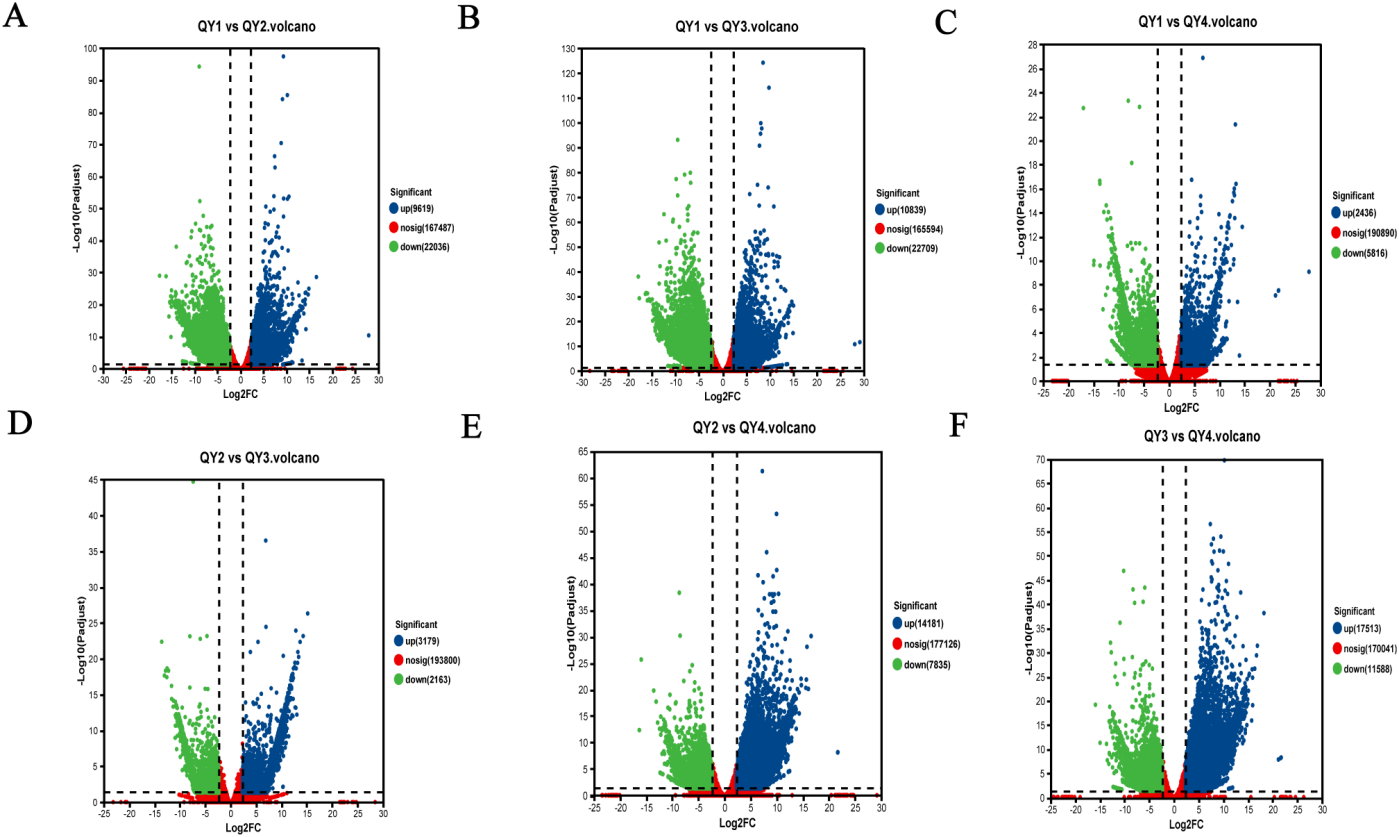
Visualization and functional characterization of differentially expressed genes (DEGs) in *G. sinensis* seeds across developmental stages. (blue/green dots represent significantly up-/down-regulated genes). **(A)** QY1 vs QY2; **(B)** QY1 vs QY3; **(C)** QY1 vs QY4; **(D)** QY2 vs QY3; **(E)** QY2 vs QY4; **(F)** QY3 vs QY4.

DEGs across the four developmental stages of *G. sinensis* seeds were visualized, and the results are presented in **Figure 3**. In this figure, abscissa represents the logarithmic value of FC (|log2(FC)|) in gene expression levels between the two samples. High absolute values indicate high fold differences in the expression levels between two samples. The ordinate represents the negative logarithm of the adjusted p-value (–log10 (Padjust)), with high values indicating more significant differential expression, thereby confirming the high reliability of the identified DEGs.

### 3.8 Cluster analysis

**Figure 4:**
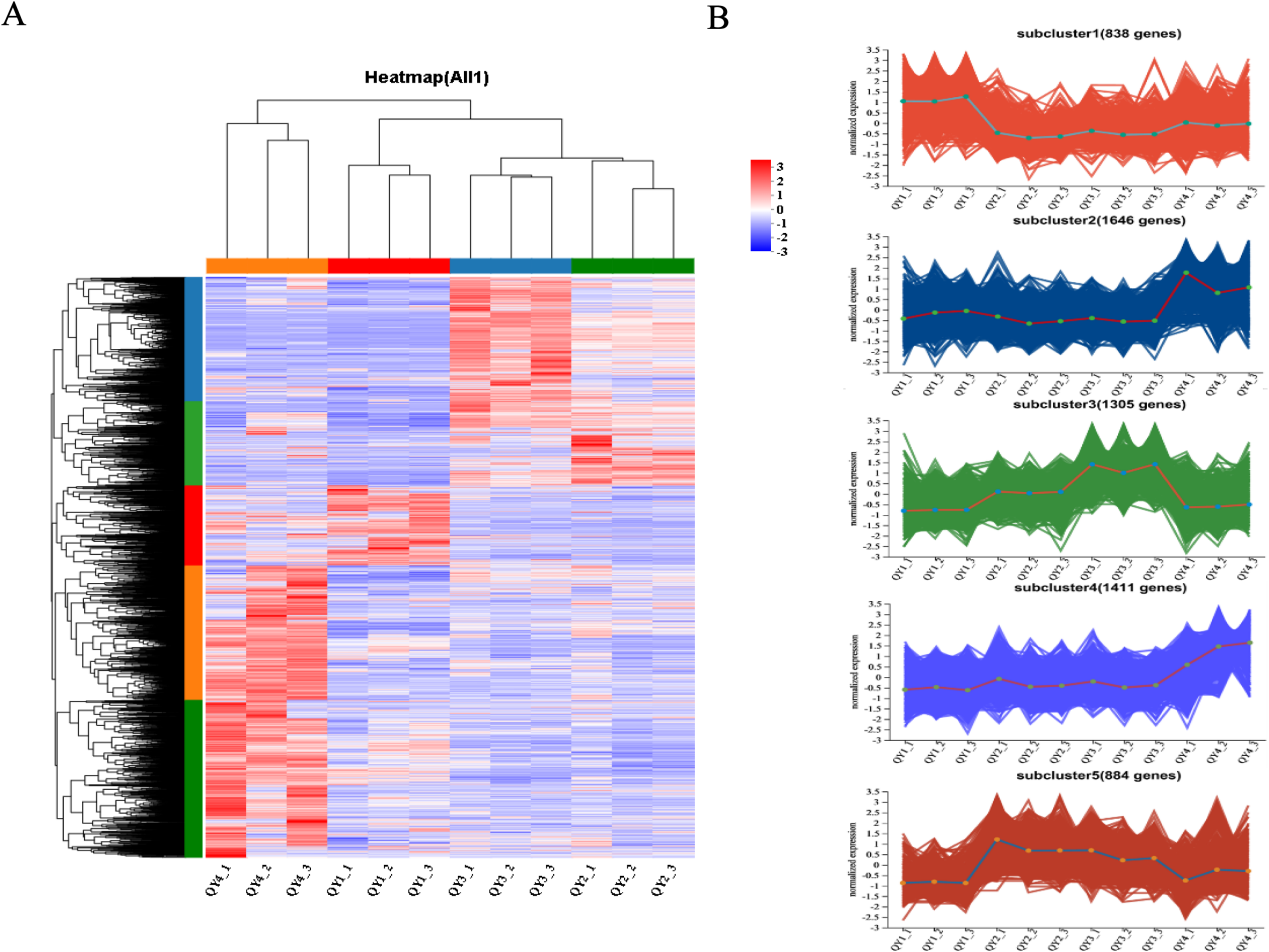
Cluster analysis of differentially expressed genes (DEGs) during *G. sinensis* seeds development **(A)** Hierarchical clustering analysis of DEGs, The color bars represent the expression levels of DEGs after TPM standardization, with red indicating high expression and blue indicating low expression; **(B)** Expression patterns of 5 subgroups of DEGs.

To understand the expression patterns of DEGs, hierarchical clustering analysis was performed for the 6,084 DEGs identified in section 3.6. As shown in **Figure 4**, all samples clustered into two main groups, with one group including the 6- and 16-week post-flowering stage samples and the other comprising the 9- and 12-week post-flowering stage samples. Clustered expression patterns of the DEG subclasses were further divided into five subgroups. Subcluster 1 was composed of 838 genes, and expression levels of these genes generally increased during the QY1 stage, suggesting that the overall expression levels of these genes are positively correlated with the early growth of *G. sinensis* seeds. In QY4, expression levels of 1,646 genes in subcluster 2 were generally upregulated. Expression levels of 1,305 genes in subcluster 3 were balanced between QY1 and QY2, increased in QY3, and decreased in QY4. Subcluster 4 contained 1,411 genes, whose expression levels gradually increased from QY1 to QY4. Expression levels of 884 genes in subcluster 5 increased during QY2 and gradually decreased from QY3 to QY4.

### 3.9 Functional annotation and enrichment analysis

**Figure 5:**
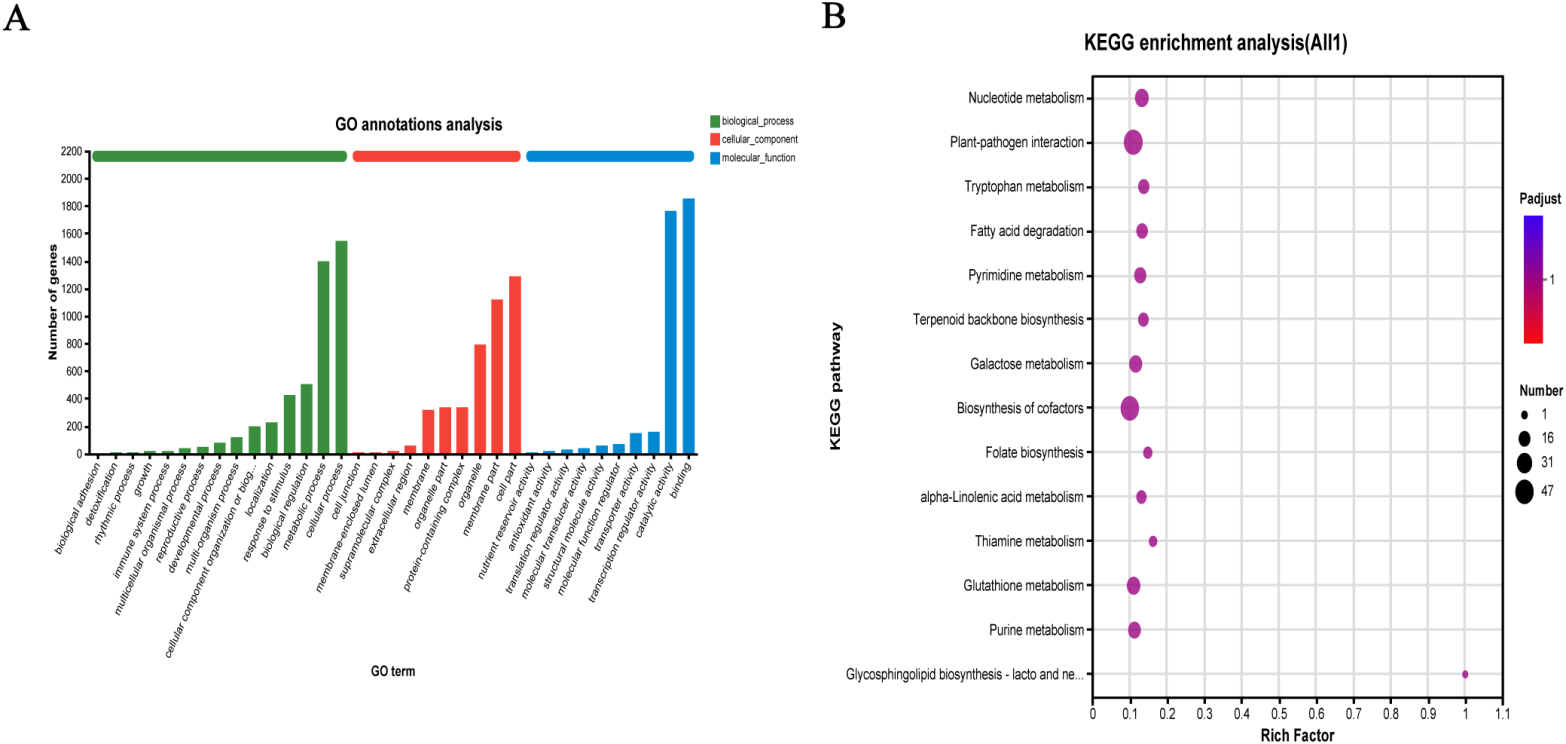
Functional annotation and pathway enrichment analysis of differentially expressed genes (DEGs) in *G. sinensis* seeds development **(A)** Gene Ontology (GO) functional classification of DEGs; **(B)** Kyoto Encyclopedia of Genes and Genomes (KEGG) pathway enrichment.

To elucidate the specific functions of the identified DEGs, GO functional annotation analysis was performed for the 6,084 DEGs in the QY1–4 samples. In the GO database, genes were classified into three main functional categories: Biological process, cellular component, and molecular function. Cellular component category included 10 subcategories, with “metabolic process,” “cell part,” and “organelle” being the top three subcategories in terms of abundance values, with 1,120 (18.41%), 1,291 (21.22%), and 793 (13.03%) genes, respectively. Biological process category included 14 subcategories, with the most annotated subcategories being “cellular process” and “metabolic process,” containing 1,545 (25.39%) and 1,396 (22.95%) genes, respectively. Of the 11 categories in the molecular function category, the two most abundant subcategories were “binding” and “catalytic activity,” with 1,856 (30.51%) and 1,762 (28.96%) genes, respectively (**Figure 5A**).

To investigate the associated metabolic pathways, 6,084 DEGs were subjected to KEGG enrichment analyses. A total of 1,466 genes were enriched in 122 biological metabolic pathways. As shown in **Figure 5B**, after setting the parameters, top 14 KEGG pathways included “nucleotide metabolism” (23 genes), “plant–pathogen interaction” (47 genes), “tryptophan metabolism” (14 genes), and “fatty acid degradation” (15 genes). Specifically, “galactose metabolism” (20 genes) and GM biosynthesis pathways were highly enriched, and “glycolysis/glycogenogenesis” (24 genes) and “other glycan degradation” (5 genes) bioinformatics pathways were also noted, providing a reference basis for further gene metabolic analysis. The top 20 candidate genes involved in galactose metabolism are listed in **Table 9**.

**Table 9:** Top 20 candidate genes involved in galactose metabolism.

Gene ID	KO_name	log_2_FC	p-adjusted
TRINITY_DN11690_c0_g2	pfkA, PFK	0.847010218	0.405360879
TRINITY_DN12146_c0_g1	E2.4.1.82	1.438784637	0.367256975
TRINITY_DN1280_c0_g1	E2.4.1.82	1.552234881	0.288405074
TRINITY_DN16183_c0_g1	E3.2.1.22B, galA, rafA	6.769086454	0.008714346
TRINITY_DN20186_c0_g1	pfkA, PFK	1.463804985	0.367070797
TRINITY_DN21111_c0_g1	E2.4.1.82	1.170273982	0.608477965
TRINITY_DN23987_c0_g1	E2.4.1.82	-0.444403564	0.841156317
TRINITY_DN24776_c0_g1	E2.4.1.82	2.74969958	0.076951355
TRINITY_DN31201_c0_g1	E3.2.1.22B, galA, rafA	0.566642575	0.770997332
TRINITY_DN35438_c0_g2	galM, GALM	3.392660345	0.317824356
TRINITY_DN35584_c1_g1	E3.2.1.22B, galA, rafA	3.298175617	0.055003143
TRINITY_DN36780_c0_g1	E2.4.1.82	-1.026833013	0.683736383
TRINITY_DN48371_c0_g1	E2.4.1.82	1.94965827	0.411083677
TRINITY_DN4929_c0_g1	HK	7.340053343	0.047291326
TRINITY_DN6099_c1_g1	E2.4.1.82	1.346792843	0.483761833
TRINITY_DN6099_c1_g3	E2.4.1.82	5.315755202	0.097647688
TRINITY_DN71347_c0_g1	GOLS	5.647175001	0.153316378
TRINITY_DN86893_c0_g1	galT, GALT	5.420290849	0.002364577
TRINITY_DN9613_c0_g1	galE, GALE	0.014037421	0.996059403
TRINITY_DN9613_c0_g2	galE, GALE	3.962347621	0.268655787

Next, genes related to lactose metabolism were analyzed to identify the genes involved in GM biosynthesis in *G. sinensis* seeds. Levels of eight enzyme genes, including fructokinase (*FK*), galactosidase (*GALA*), galactose gyrase (*GALM*), hexokinase (*HK*), inositol galactoside synthase (*GolS*), phosphogalactosyltransferase (*GALT*), UDP galactose-4-isomerase (*GALE*), and raffinose synthase (*RS*), increased at different growth stages, indicating that these genes were related to the biosynthesis of GM, the main component of *G. sinensis* endosperm.

When conducting conservative motif analysis on the eight key genes involved in GM synthesis selected from transcriptome data and related genes in *Arabidopsis thaliana*, *Zea mays* and *Glycine max*, we obtained highly valuable results. Eight genes exhibit conserved motif features similar to homologous genes in *A. thaliana*, *Z. mays* and *G. max*, which to some extent reflects their evolutionary conservation and potential universal biological functions in GM synthesis. This provides some support for the identity of these genes as key regulatory factors at the sequence level (**Supplementary Figure 11**).

### 3.10 Prediction of GM biosynthesis-related genes

**Figure 6:**
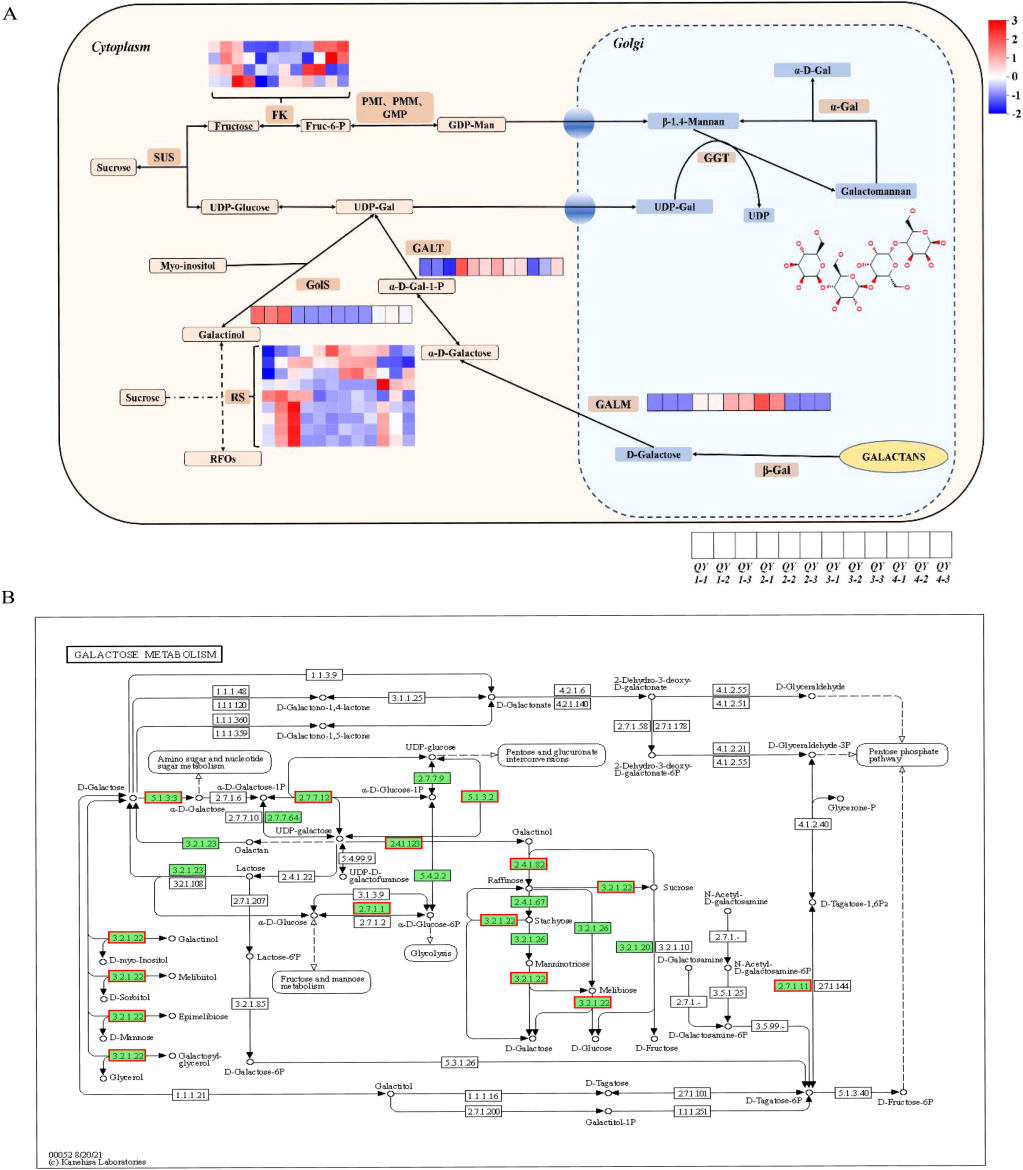
GM biosynthesis pathway prediction. **(A)** GM biosynthesis pathway prediction model diagram. Heatmap colors represent the logarithmic values of gene expression differences among different samples, with red indicating upregulation and blue indicating downregulation. **(B)** KEGG enrichment analysis of the galactose metabolism signaling pathway annotation diagram. Green background indicates the transcript obtained via sequencing, and red border indicates the upregulation of expression levels.

After enriching and analyzing the DEGs using relevant databases, 20 unigenes were found to be involved in galactose metabolism, expressing eight related enzymes, including FK, GALA, GALM, HK, GolS, GALT, GALE, and RS. Analysis of transcript expression in different growth stages revealed the upregulation of all eight factor levels. By combining transcriptome-related data with the GM biosynthesis model proposed by Joët, Akutsu, Gaikwad, Hu, and Wang et al (Wang et al., 2012; Joët et al., 2014; Hu et al., 2019; Gaikwad et al., 2023; Akutsu et al., 2024), a visual heatmap of the inferred GM biosynthesis pathway was constructed to visualize the dynamic changes in gene expression levels during *G. sinensis* seed development. This pathway is illustrated in **Figures 6A, B**.

Under the action of invertase, sucrose is converted to fructose and glucose, which subsequently participate in GM synthesis through two distinct metabolic pathways (Shivanna et al., 1989). Fructose is phosphorylated by *FK* to form fructose-6-phosphate, which is converted to GDP-mannose in the presence of PMI, PMM, and GMP. UGE catalyzes the conversion of UDP-glucose to UDP-galactose. Substrates, such as GDP-mannose and UDP, are synthesized in the cytoplasm and subsequently transported to the Golgi apparatus by sugar nucleotide transporters for GM biosynthesis (Seifert, 2004). *ManS* used GDP mannose to generate a mannose polysaccharide backbone, and galactose residues are transferred from UDP-galactose to the mannose backbone via GGT, resulting in GM synthesis (Dhugga et al., 2004).

Gossypose is a raffinose family oligosaccharide (RFO) stored in the vacuoles of legume endosperm that is primarily responsible for the transient accumulation of carbohydrates. Through this accumulation, UDP galactose and GDP mannose are continuously replenished, forming GM (Joët et al., 2014). These results suggest that GM biosynthesis pathway in *G. sinensis* gum is inter-regulated with the RFO metabolic pathway.

By integrating the predicted model of the GM biosynthesis pathway, we found that the key regulatory factors (*FK*, *RS*, *GolS*, *GALM*, and *GALT*) were all expressed in the different growth stages of *G. sinensis* seeds. Notably, *RS* was active during the QY1–QY4 growth stages and contained nine gene copies. *FK* had four gene copies, whereas *GolS*, *GALM*, and *GALT* each had one gene copy. *GolS* levels were upregulated at the QY1 stage, *GALT* levels at the QY2 stage, *GALM* levels at the QY3 growth stage, and *FK* levels at both the QY1 and QY4 growth stages, indicating that *RS*, *FK*, *GolS*, *GALM*, and *GALT* play crucial regulatory roles in the different growth stages of *G. sinensis* seeds. This suggests that GM biosynthesis in *G. sinensis* polysaccharide gum is regulated by the RFO metabolic pathway, consistent with the previously proposed theory of GM formation in leguminous plants (Singh et al., 1990; Tyagi et al., 2018; Hu et al., 2019; Gaikwad et al., 2023; Akutsu et al., 2024).

## 4 Discussion

*G. sinensis* seeds contain abundant polysaccharides, which can be isolated and extracted from the seed endosperm to obtain the polysaccharide gum. A 1% aqueous solution of polysaccharide gum exhibits better apparent viscosity, natural biodegradation resistance, and stability than guar gum. It is used as a thickener, adhesive, and stabilizer in various industries, such as the food, petroleum, papermaking, printing and dyeing, and mineral processing industries (Liu, 2012; Liu et al., 2017). Structurally, *G. sinensis* polysaccharides are similar to the commercially used guar and locust bean gums and belong to the GM category. To better understand the GM biosynthesis pathway and regulatory mechanisms, we isolated RNA from the endosperm of *G. sinensis* seeds at four different developmental stages and constructed a cDNA library in this study. GM content was determined via HPLC. Based on the results, QY samples were selected for subsequent transcriptome analysis, which generated 79.13 GB of clean data, 19,914 transcripts, and 110,141 unigenes. By annotating and aligning the assembled unigenes against six major databases, we obtained 44,378 (GO), 18,662 (KEGG), 24,197 (Protein Family), 31,740 (Swiss-Prot), 41,792 (eggNOG), and 53,262 (NR) annotated results. Based on the pairwise comparisons of gene expression levels at the four growth stages, 10,291 DEGs, including 4,710 upregulated and 5,581 downregulated DEGs, were identified. Among these, 6,084 candidate genes were selected for further analysis via clustering analysis and Venn diagram visualization of DEGs. Functional annotation and enrichment analysis of these candidate genes using the GO and KEGG databases revealed that eight enzyme genes (*FK*, *GALA*, *GALM*, *HK*, *GolS*, *GALT*, *GALE*, and *RS*) were involved in the GM biosynthesis pathway and positively regulated GM biosynthesis.

Three enzymes and their coding genes (*ManS*, *GMGT*, and *α-Gal*) essential for GM synthesis have been isolated and functionally characterized in other plants (Joët et al., 2014; Akutsu et al., 2024). Wang et al. (2012) identified a group of genes encoding GM biosynthesis-related enzymes (*PMI*, *PMM*, *GGT*, *ManS*, *UGE*, *GMP/MGT*, *FK*, and invertase), and Hu et al. (2019) reported another group of genes (e.g., *GALK*, *b-Gal*, *GALM*, and *GALT*) highly expressed in the endosperm and suggested the involvement of these genes in GM metabolism regulation. Levels of transcription factors NAC and MYB, the main regulatory factors activating secondary cell wall biosynthesis, are upregulated in fenugreek endosperm (Zhong et al., 2010; Ambavaram et al., 2011). Tyagi et al. reported three novel microRNAs (*CtmiR-3157*, *Ct-miR3130*, and *Ct-miR1315*) as important regulatory factors in guar seeds (Tyagi et al., 2018). Consistent with previous reports, *FK*, *GALM*, and *GALT* were also identified in this study, confirming the reliability of our transcriptome sequencing results.

Sorbitol and RFOs, such as raffinose and raffinose, are transiently accumulated during endosperm development, serving as temporary storage carbohydrates for GDP mannose and UDP galactose synthesis (Joët et al., 2009). In addition to these factors, endosperm GM composition is also influenced by the climate. Low temperatures favor RFO and sorbitol synthesis but delay endosperm GM biosynthesis, indicating the close relationship between GM, RFO, and sorbitol metabolism during seed development (Joët et al., 2014).

As shown in the inferred GM biosynthesis pathway diagram in **Figure 6A**, *FK*, *GALM*, *GolS*, *GALT*, and *RS* were involved in the regulation of GM biosynthesis; however, *HK*, *GALA*, and *GALE* could not be unidentified. RFO metabolism is involved in multiple physiological processes, including plant transport, abiotic stress resistance, and seed vigor development. Specifically, RS is an important initiating factor in the RFO metabolic pathway that synthesizes or converts sucrose step-by-step into the components required for other physiological activities. In this study, RS exhibited the highest number of gene copies, indicating that the metabolic pathways of GM biosynthesis and RFOs are mutually regulated during the growth and development of *G. sinensis*. However, this study only analyzed the expression levels of these genes, warranting further investigations to verify their functions.

Similar to the biosynthesis of other plant wall polysaccharides, GM biosynthesis in *G. sinensis* seeds is a complex process involving multiple reactions in different cellular compartments (Seifert, 2004). In addition to *FK*, *GALM*, *GolS*, *GALT*, and *RS*, GM biosynthesis in *G. sinensis* seeds requires the coordinated action of several enzymes and transporter proteins. Therefore, identification of genes encoding these related proteins is essential to understand the biosynthesis pathways and regulatory mechanisms of GM. Future studies should use next-generation sequencing technology (454 sequencing) for in-depth expressed sequence tag analysis to identify the additional genes involved in GM biosynthesis and regulation.

Despite identifying the key candidate genes involved in the biosynthesis of *G. sinensis* seed GM (*FK*, *GALM*, *GolS*, *GALT*, and *RS*) via RNA sequencing analysis, this study was limited by the lack of adequate experimental validation (e.g., reverse transcription-quantitative polymerase chain reaction and enzyme activity assays) due to difficulties related to the experimental conditions (e.g., sample unavailability and insufficient technical resources) and constraints in research time and funding. Nevertheless, our results are consistent with the GM formation theory in leguminous plants of Singh et al., supporting the reliability of our transcriptome data (Singh et al., 1990; Tyagi et al., 2018; Hu et al., 2019; Gaikwad et al., 2023; Akutsu et al., 2024). Future studies should validate the functions of the identified genes via genetic and *in vitro* testing.

The latest research mainly reports the *de novo* assembly of the chromosome level genome of *G. sinensis* and reveals its evolutionary process and molecular mechanisms of thorn development (Xiao et al., 2025). Although the article did not directly mention GM synthesis research, some limitations and future directions regarding plant genetic engineering and molecular breeding can be inferred from it. Although transcriptomics plays an important role in studying gene expression and regulation, relying solely on transcriptomics to study key regulatory genes in the GM synthesis pathway has the following limitations: transcriptomics provides a snapshot of gene expression at a specific time point and can not capture the dynamic changes in gene expression processes; Transcriptomics can only display the correlation of gene expression, but cannot directly prove causal relationships. Although transcriptomics provides important information in studying GM, its limitations cannot be ignored. Future research should combine other omics techniques (such as proteomics, metabolomics, etc.) and functional validation experiments to gain a more comprehensive understanding of the regulatory mechanisms of GM synthesis.

## 5 Conclusion

To the best of our knowledge, this study is the first to construct 12 cDNA libraries of *G. sinensis* seeds using the Illumina HiSeq 4000 sequencing platform. The predicted GM biosynthesis pathway model revealed five genes (*FK*, *GALM*, *GolS*, *GALT*, and *RS*) encoding key enzymes in the GM biosynthesis pathway, facilitating further genomic research on *G. sinensis* seeds and cloning and functional analysis of GM biosynthesis-related enzymes. Furthermore, our transcriptome sequencing approach can contribute to future genetic and genomic research on the chemical constituents of *G. sinensis* seeds and improvement of their active ingredient levels via genetic engineering.

## Data Availability

All sequencing data have been deposited into the National Center for Biotechnology Information (NCBI) Sequence Read Archive (SRA) database under accession number PRJNA1242246.
