# Supplementary material for: Transcriptomic reveals key genes and regulatory pathways in galactomannan biosynthesis in Gleditsia sinensis polysaccharide gum
Source: Front Plant Sci. 2025 Jul 23;16:1555374. doi: 10.3389/fpls.2025.1555374 (PMC12325253; doi:10.3389/fpls.2025.1555374)
Supplement: Supplementary file 1 [file DataSheet1.docx]

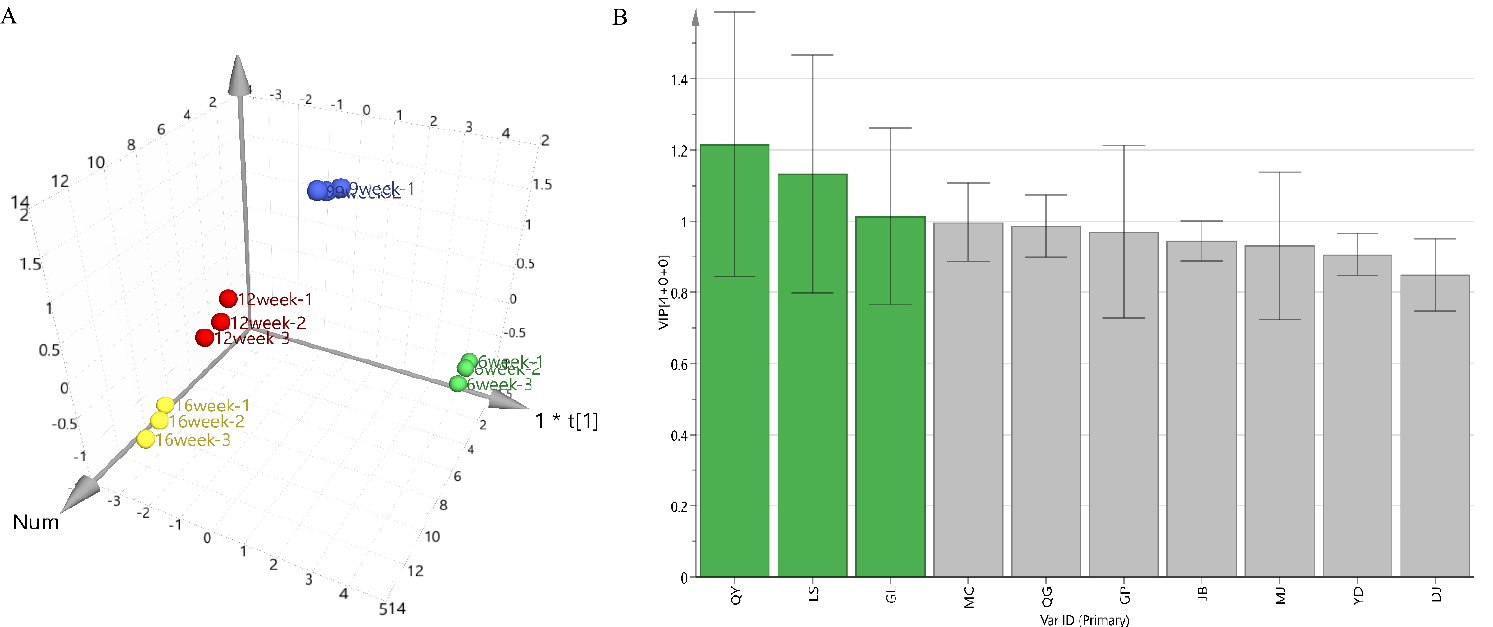


Supplementary Figure 1. Orthogonal partial least-squares discriminant analysis (OPLS-DA). (A) OPLS-DA model diagram. (B) Differential variable importance in projection (VIP) value graph.


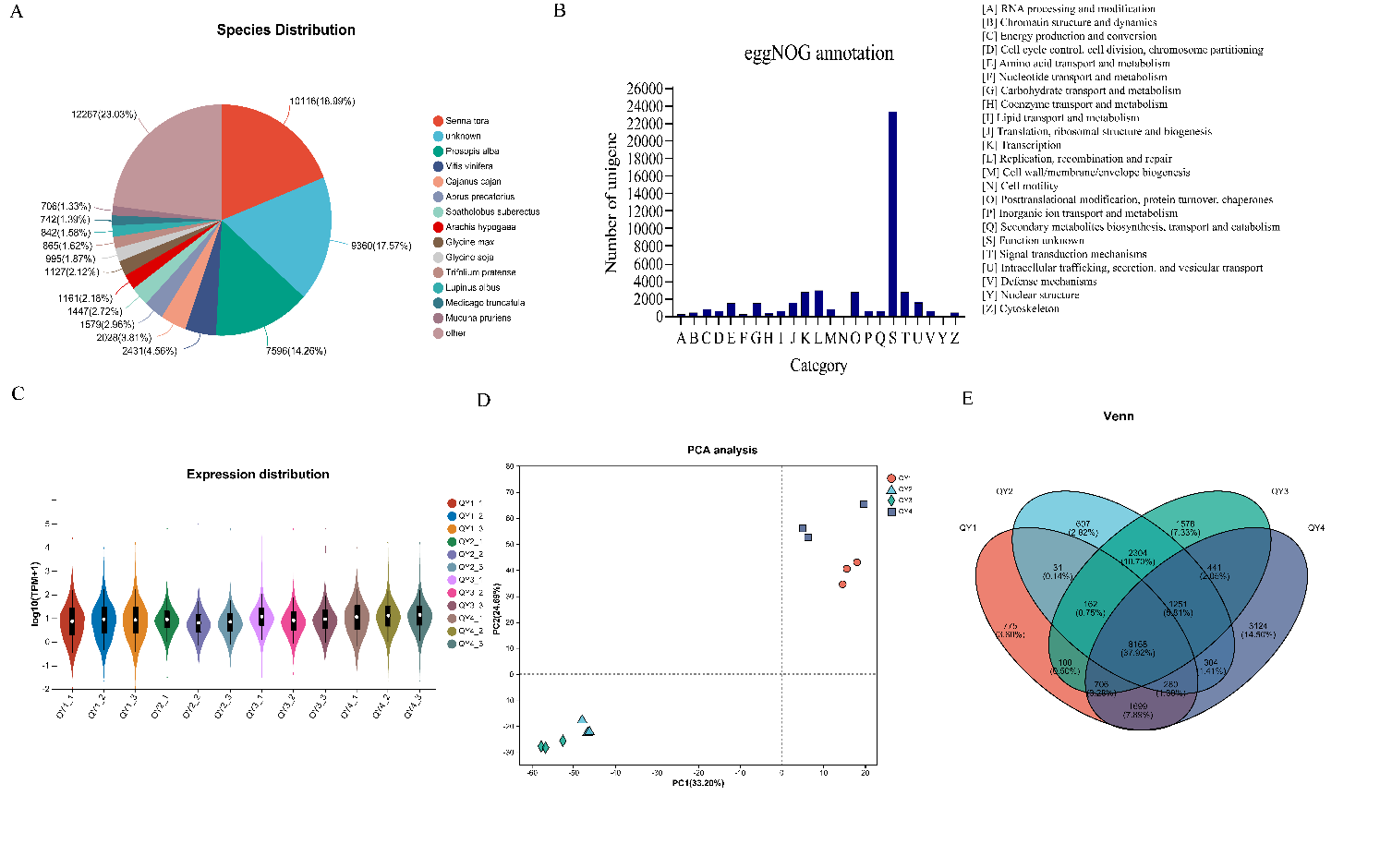


Supplementary Figure 2. Functional annotation and gene expression analysis. (A) Gene Non-Redundant (NR) annotation species distribution. (B) Gene eggNOG functional annotation. (C) Expression level distribution. (D) Principal component analysis (PCA) of G. sinensis seed samples. (E) Venn analysis of samples.


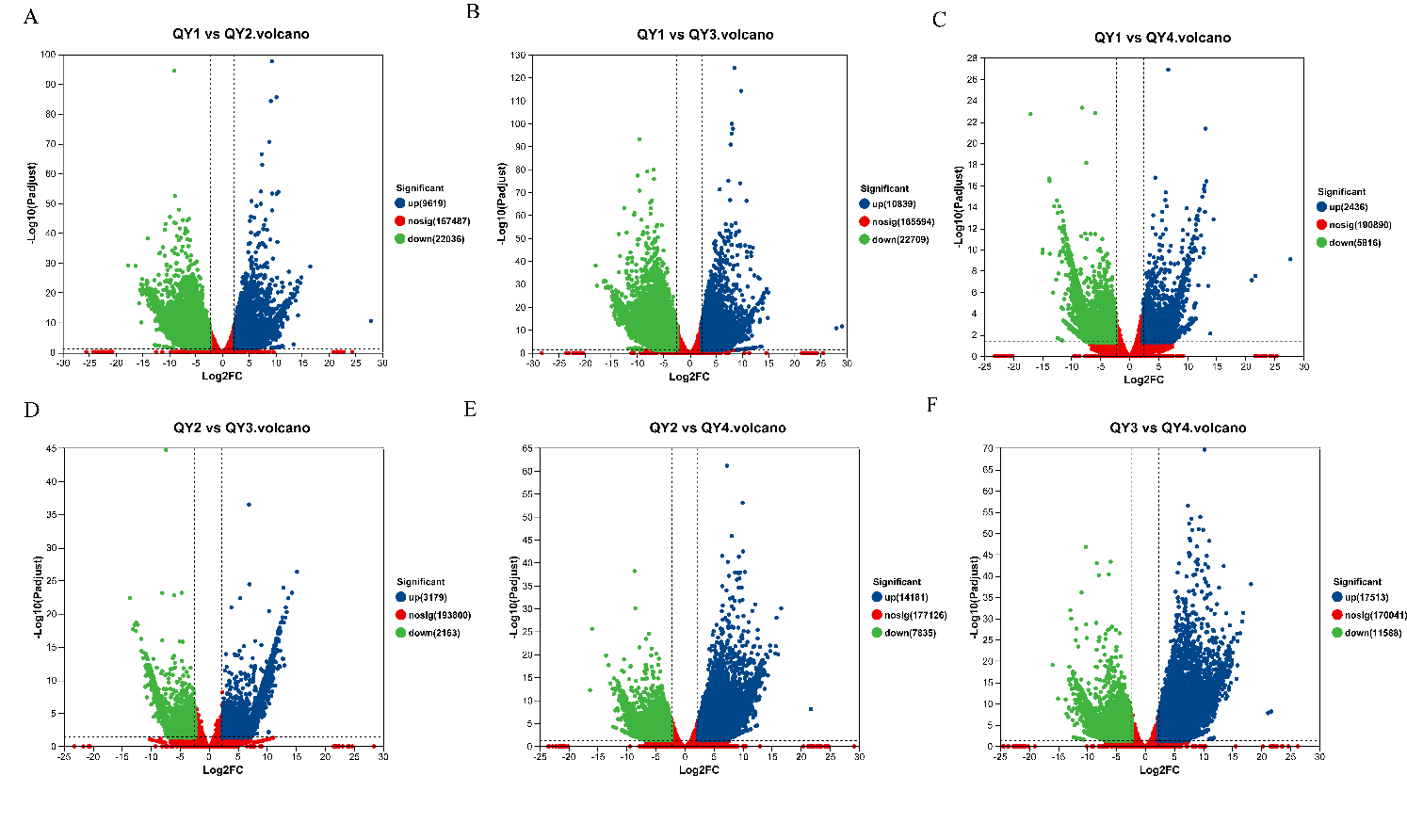


Supplementary Figure 3. Visualization of the differentially expressed genes (DEGs) in Gleditsia sinensis seeds at different growth stages.


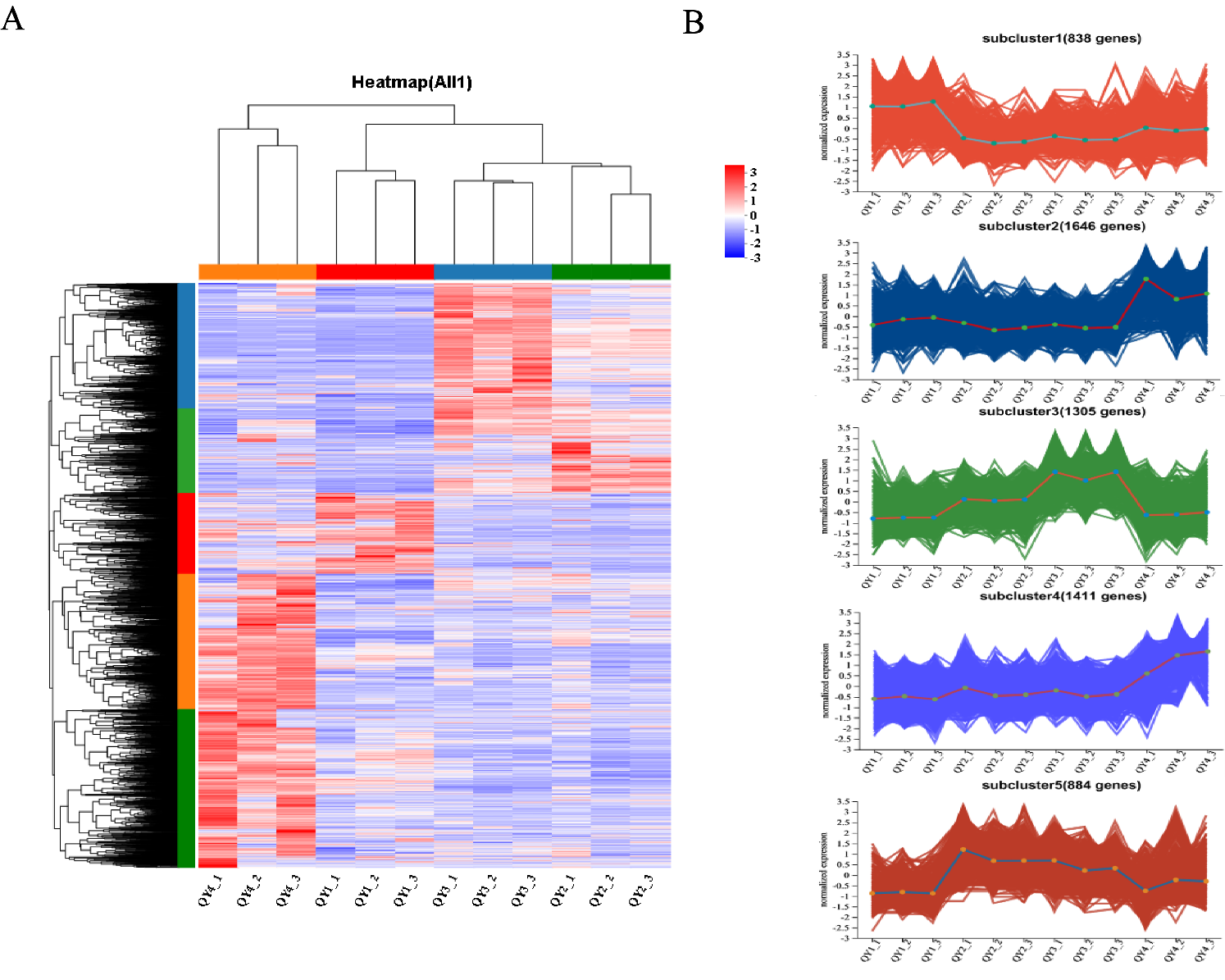


Supplementary Figure 4. Cluster analysis of DEGs. (A) Hierarchical clustering analysis of DEGs. Color bars represent the DEG expression levels after transcripts per million (TPM) standardization, with red indicating high levels and blue indicating low levels. (B) Expression patterns of five DEG subgroups.


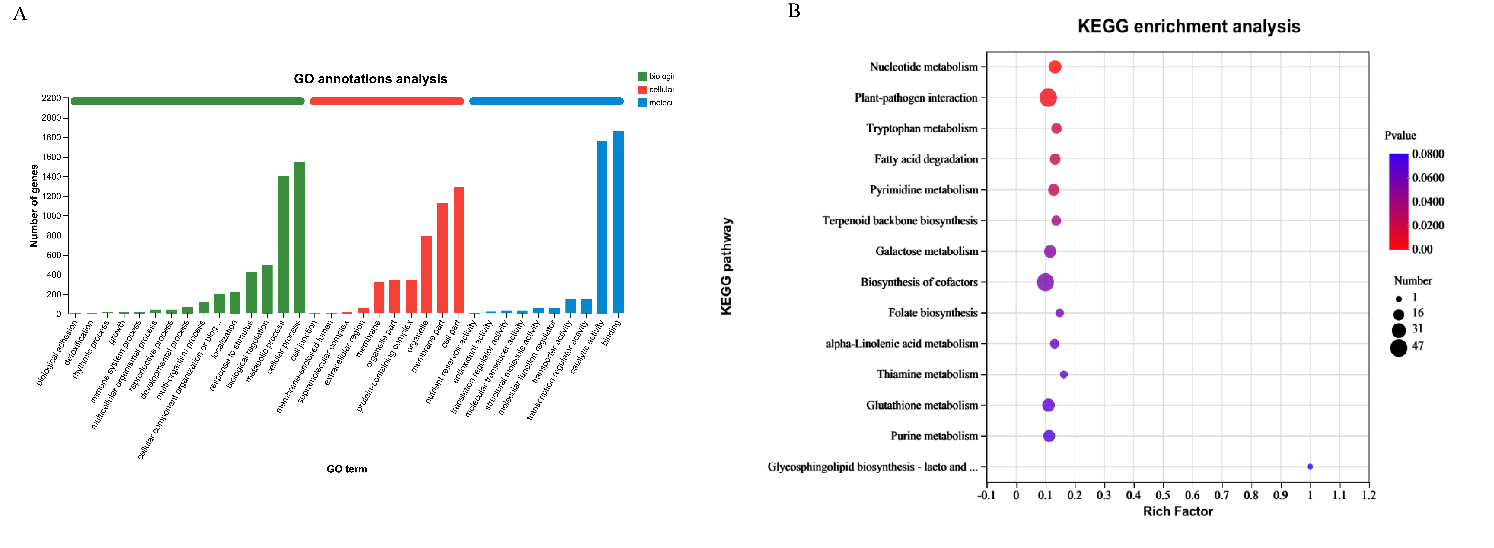


Supplementary Figure 5. Functional annotation and enrichment analysis of DEGs. (A) Differential Gene Ontology (GO) functional annotation. (B) Kyoto Encyclopedia of Genes and Genomes (KEGG) enrichment analysis.


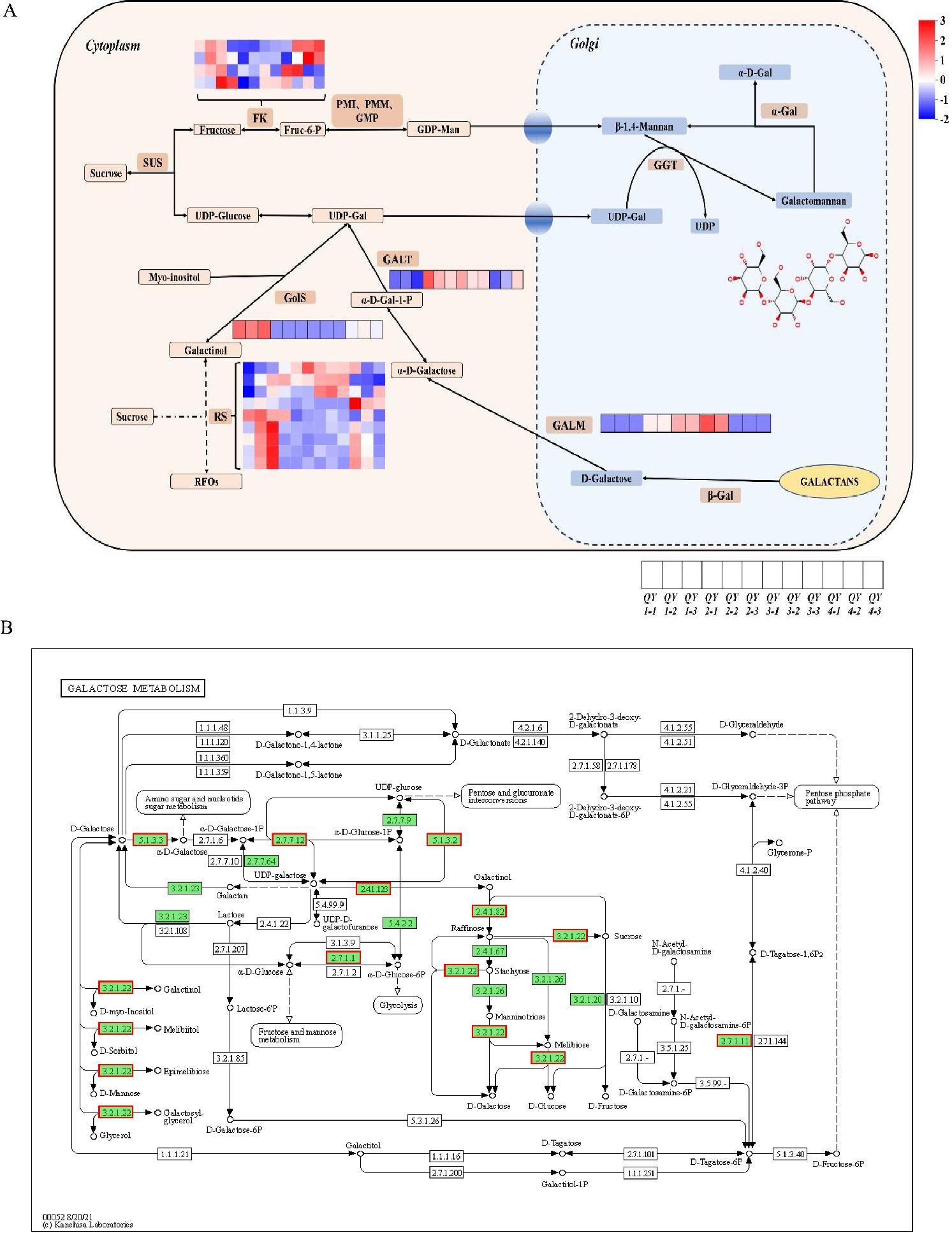


Supplementary Figure 6. Galactomannan (GM) biosynthesis pathway prediction. (A) GM biosynthesis pathway prediction model diagram. Heatmap colors represent the logarithmic values of gene expression differences among different samples, with red indicating upregulation and blue indicating downregulation. (B) KEGG enrichment analysis of the galactose metabolism signaling pathway annotation diagram. Green background indicates the transcript obtained via sequencing, and red border indicates the upregulation of expression levels.


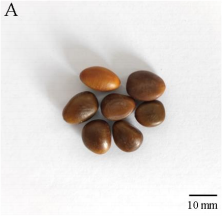


## Supplementary Figure 7. *G. sinensis* Lam. Seeds


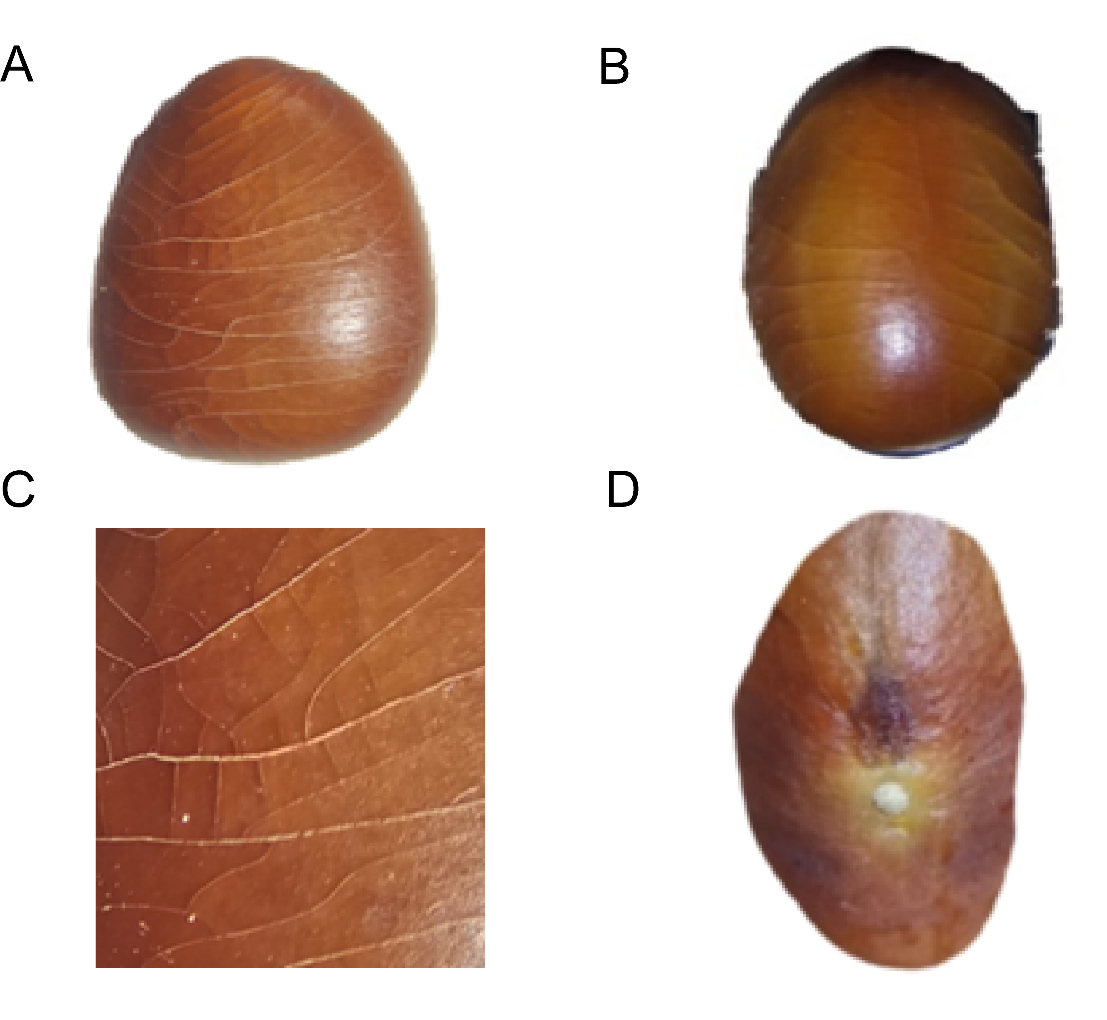


**Supplementary Figure 8.** Appearance diagram of *G. sinensis* Lam. Seeds; (A) Front View; (B) Side View;(C) Surface View;(D) Umbilical View


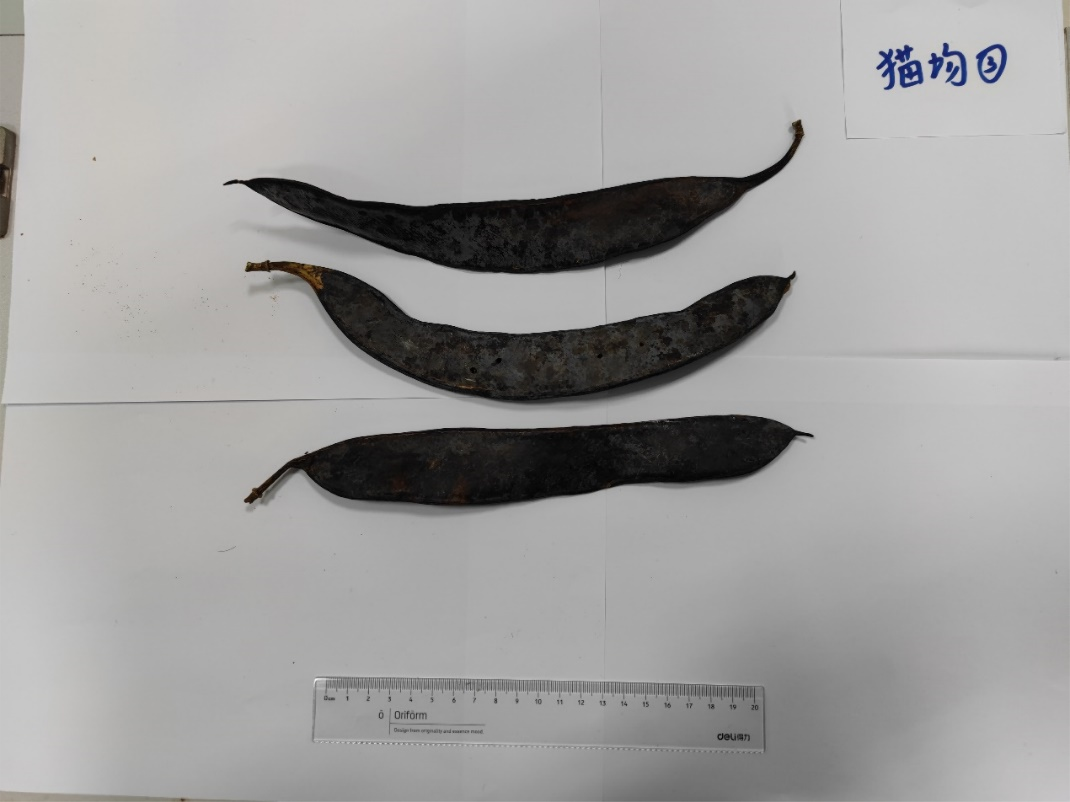


**Supplementary Figure 9.** Fruit of *G. sinensis* Lam


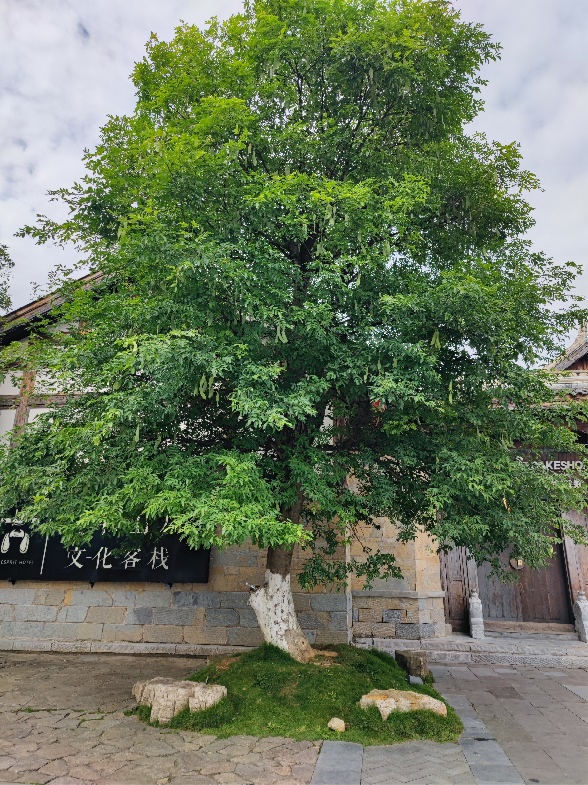


**Supplementary Figure 10.** *G. sinensis* Lam plant


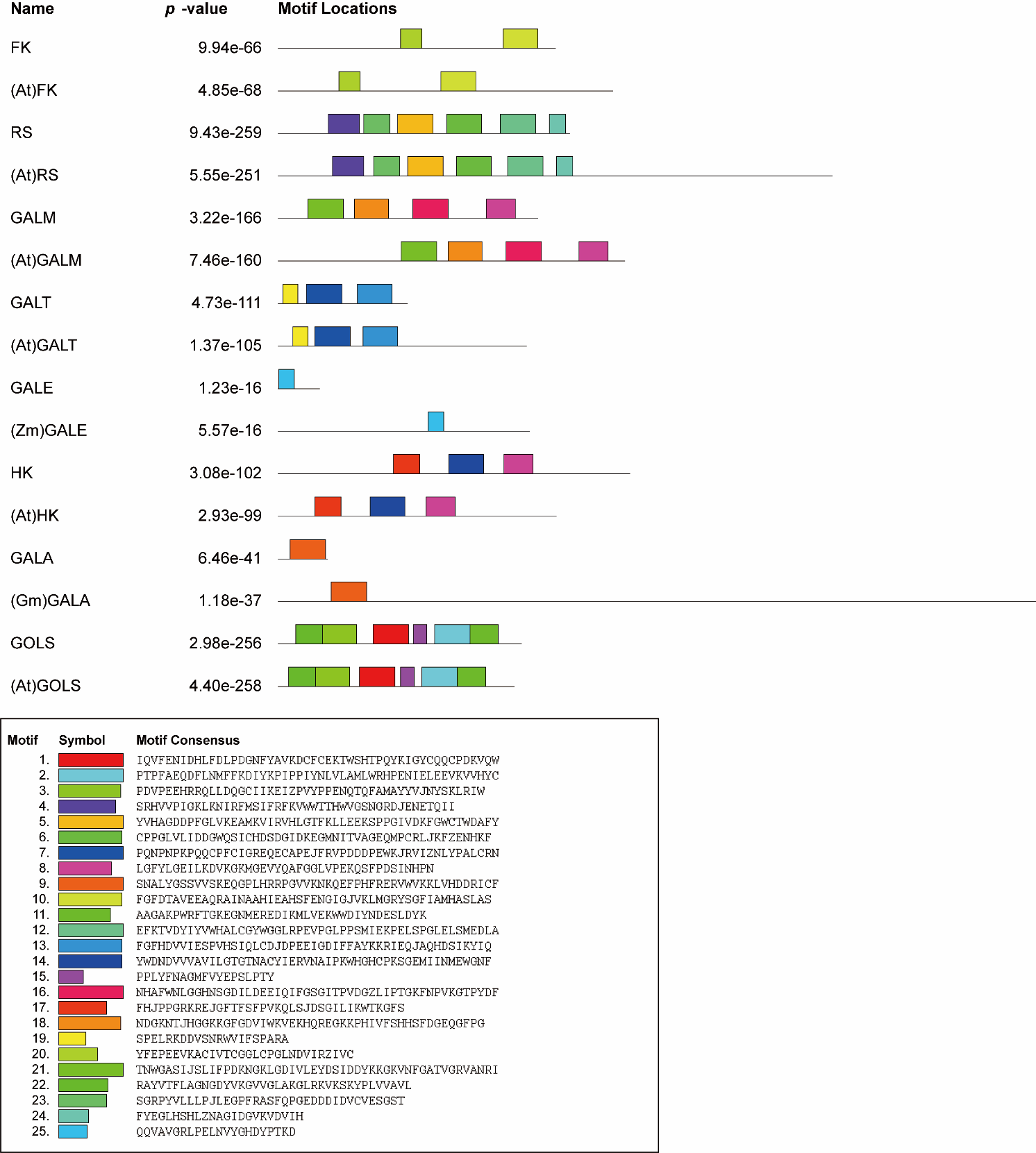


**Supplementary Figure 11.** Conserved motifs of *FK*、*RS*、*GALM*、*GALT*、*GOLS*、*HK*、*GALA*、*GALE*
